# Prevalence of Diabetes Mellitus among Patients with Tuberculosis and Its Associated Factors in Sana’a, Yemen, 2021

**DOI:** 10.3390/epidemiologia4020021

**Published:** 2023-06-13

**Authors:** Sumia Alturki, Mohammed Al Amad, Esam Mahyoub, Noora Al Hanash, Abdulbary Alhammadi

**Affiliations:** 1 Yemen Field Epidemiology Training Program, Ministry of Public Health and Population, Sana’a City, Yemen; 2 Yemen National Tuberculosis Center Program, Ministry of Public Health and Population, Sana’a City, Yemen

**Keywords:** tuberculosis, TB–DM, national tuberculosis center, Yemen

## Abstract

Diabetes mellitus (DM) is one of tuberculosis’ (TB) ending barriers. TB patients with DM are at a higher risk than non-diabetes patients to develop complication, relapse and die. In Yemen, information on TB–DM comorbidity is lacking. This study aimed to determine the prevalence and associated factors of diabetes among TB patients at the National Tuberculosis Center (NTC) in Sana’a. A facility-based cross-sectional study was conducted. All TB patients aged >15 years who attended the NTC from July to November 2021 were screened for DM. Socio-demographic and behavioral information were collected through face-to-face interviews using questionnaires. A total of 331 TB patients were enrolled, 53% were males, 58% aged <40 years, and 74% were newly diagnosed with TB. Overall, DM prevalence was 18%. Higher rates of DM were found among TB patients that were male (OR = 3.0; 95% CI; 1.4–6.7), ≥50 years of age (OR = 10.8; 95% CI; 4.3–27.3), and those with a family history of diabetes (OR = 3.4; 95% CI; 1.6–6.9). Almost one fifth of TB patients had DM. The early detection of DM through immediate screening after a TB diagnosis and periodically during the course of treatment is crucial for TB patients’ optimal care. Dual diagnostics for reducing the dual burden of TB–DM comorbidity is recommended.

## 1. Introduction

Tuberculosis (TB) is a chronic infectious disease caused by the bacteria Mycobacterium tuberculosis [1]. The World Health Organization (WHO) post-2015 End TB Strategy aims to end the global TB epidemic as part of its newly adopted Sustainable Development Goals [2]. Between 2000 and 2020, an estimated 66 million lives were saved through TB diagnosis and treatment [2]. In 2020, an estimated 10 million people developed TB and 1.5 million died with TB [3,4].

The increasing burden of non-communicable diseases, particularly diabetes mellitus (DM), presents a threat to the progress made towards ending TB [5,6]. Individuals with diabetes are three times as likely to develop TB compared to those who do not have diabetes [7]. Thus, 5% of global TB cases are attributed to DM [6]. The prevalence of DM among TB patients ranged from 1.8% to 45% in low- and middle-income countries [3,8,9]. TB patients with DM have a higher risk of treatment failure, relapse and an accelerated emergence of TB drug-resistance [3,10]. TB patients with DM are almost four times as likely to experience a relapse of their TB after being cured, nearly twice as likely to develop multidrug resistance, and twice as likely to die during TB treatment, compared to TB patients without DM [7,11].

Many studies have been carried out to measure the magnitude of TB–DM comorbidity. In India, nearly 50% of TB patients had either diabetes or pre-diabetes [12]. Previous studies showed variable prevalence rates of DM among TB patients, ranging from 5% to 41.1% [13,14,15,16,17]. In Yemen, TB is the fourth leading cause of death, with an estimated incidence rate of 48 per 100,000 members of the population in 2017 [14]. The epidemiology of DM in Yemen remains unclear [13], and the information on the magnitude of TB–DM comorbidity is still lacking. This study aimed to determine the prevalence of DM and its associated factors among TB patients at the National Tuberculosis Center (NTC), Sana’a-Yemen, in 2021.

## 2. Materials and Methods

### 2.1. Study Design

A facility based cross-sectional study was conducted in the NTC, a referral TB center in Sana’a city, during July–November 2021.

### 2.2. Study Area and Setting

The NTC is one of four centers under the National Tuberculosis Control Program (NTCP) in Sana’a capital city and is the national reference health facility for TB. Its primary responsibility is to ensure that TB control is implemented effectively and uniformly throughout the country. It is within the jurisdiction of the Ministry of Public Health and Population (MoPHP), under the guidance and supervision of the Director General of Public Health Care and the Under-Secretary of Medical Services and Primary Health Care.

### 2.3. Study Population

All patients with TB aged more than 15 years who were diagnosed with TB and registered at the NTC during the study period were included in this study. Patients were included in this study if they met the case definition for TB. Pulmonary tuberculosis (PTB) cases were defined as patients with a positive bacteriological culture (solid or liquid), a sputum-smear test or a positive molecular test. Extra-pulmonary tuberculosis (EPTB) cases were defined as cases confirmed microbiologically either by nucleic acid tests, a positive smear for acid fast bacilli (AFB), or an automated liquid culture [15]. Clinically diagnosed patients were defined as patients with negative microbiological tests for TB, but strong clinical suspicion and other evidence of EPTB, such as compatible imaging findings, histopathological findings, ancillary diagnostic tests or responsiveness to anti-TB treatment as in an EPTB case [15].

Inclusion Criteria: TB patients who met the case definition; registered or new TB patients at the NTC; and TB patients who consented to participate in this study.

Exclusion Criteria: TB patients less than 15 years old; pregnant/lactating mothers; and all TB patients who did not consent to participate in this study.

### 2.4. Sample Size

Based on power and sample size calculations, a sample of 322 patients is needed to estimate the prevalence of DM at 30%, with a 5% margin of error using an alpha level of 0.05v and a power of 80%.

### 2.5. Date Collection

Data were collected by well-trained health workers. A structured questionnaire was used to collect sociodemographic data (age, sex, education, occupation and place of residence) and behavioral data (smoking, chewing khat, family history of diabetes, food habit, type of TB and chronic diseases) through face-to-face interviews, while TB diagnostic data were extracted from patients’ cards. Anthropometric data (height and weight) were measured. Weight was measured while the patient wearing light clothes to the nearest 0.5 kg. Height was measured to the nearest 0.5 cm. Laboratory testing was performed for fasting blood glucose (FBS), random blood glucose (RBS) and glycated hemoglobin (HbA1C).

Human samples and laboratory procedures: 3 mL of blood was drawn by well-trained laboratory technicians and collected into two tubes (plane and EDTA tubes). The samples were labeled and processed immediately. Serum was separated from the blood via centrifuging. Glycosylated hemoglobin (HbA1c) was measured by using RESPONS 940, an automated random-access clinical chemistry analyzer; fasting and random blood glucose were measured by a standardized glucometer (TMW MEDICAL).

### 2.6. Date Management

Diabetes was diagnosed based on WHO guidelines: patients self-reported having diabetes and were on diabetes treatment, had fasting blood glucose levels ≥ 126 mg/dL, random blood glucose levels ≥ 200 mg/dL, or glycated hemoglobin (HbA1c) levels ≥ 6.5% [16]. Body mass index (BMI) was calculated as weight in kg divided by height in meter squared^2^. BMI was categorized according to WHO criteria [17]: underweight, <18 kg/m^2^; normal weight, 18–22.9 kg/m^2^; overweight, 23–24.9 kg/m^2^; and obese, >25 kg/m^2^.

### 2.7. Date Analysis

Epi-Info version 7.2 was used for data entry and analysis. Chi-square (X^2^) was used to test differences in categorical variables. Univariate and multivariate binary logistic regressions were used to calculate the prevalence odds and determine the factors associated with diabetes among TB patients. *p*-value ≤ 0.05 was considered as the cut off point for statistical significance, along with a 95% Confidence Interval (CI).

### 2.8. Ethical Considerations

Ethical clearance for this study was submitted to the ethics committee at MoPHP for approval (ID:111, 2021) and permission from the NTCP was given to conduct this study. TB patients aged 15 years and above were this study’s participants. Both written and verbal consent were obtained from all individual participants included in this study before the questionnaire was filled in and before the blood samples were collected. The written consents obtained were kept in individual patient files and were documented. New diabetic patients detected by the screening were referred to specialized clinics for additional management. Lab results were given to all participates, and all information obtained from the patients were kept confidential.

## 3. Results

### 3.1. Participants Characteristics

A total of 331 TB patients at the NTC were enrolled in our study. Almost half, 53%, were males, 58% were aged < 40 years, 52% were from rural area, 44% were illiterate, and 86% did not work. Smokers made up 23% and 61% were khat chewers (a mildly narcotic herb). Regarding clinical characteristics, 57% had EPTB, and 43% had PTB. For nutritional status, 34% were underweight and 25% had comorbidity with chronic diseases (14% heart diseases and 9% kidney diseases). See Table 1 for the sociodemographic and clinical characteristics of TB patients, National Tuberculosis Center, Sana’a-Yemen 2021.

### 3.2. Prevalence of Diabetes among TB Patients

Out of 331 patients, 58 (18%) were diabetic according to the WHO definition. A total of 24 (7%) were newly diagnosed and 34 (11%) were diabetics and known for their diabetic status. See Table 2 for the diabetes status among TB patients at the National Tuberculosis Center, Sana’a-Yemen 2021.

Out of 58 TB–DM patients, 67% were males, 66% were aged ≥ 50 years, and 60% were illiterate. Smoking and khat chewing behaviors accounted for 28% and 60% of TB–DM patients compared to 22% and 61% among non-diabetes patients, respectively. See Table 3 for characteristics of TB–DM with TB patients, National Tuberculosis Center, Sana’a-Yemen 2021.

### 3.3. Factors Associated with Diabetes Prevalence

The results of the univariate binary logistic regression shows that the odds of males with TB having diabetes is 2.1 times (95% Cl; 1.2–3.8) than females; patients aged ≥50 years old are more likely to have diabetes by 7.5 times (95% Cl; 4.1–14.1) than those <50 years of age. Uneducated patients are more likely to have diabetes than educated TB patients (POR 2.2, 95% Cl; 1.2–3.9). Compared with the relevant groups, patients with a family history of diabetes (POR 2.3, 95% Cl; 1.3–4.1) and obese patients (POR 2.3, 95% Cl; 1.1–5.0) are more likely to have diabetes. See Table 4 for the univariate analysis for factors associated with diabetes prevalence among TB patients, National Tuberculosis Center, Sana’a-Yemen 2021.

In the multivariate analysis, only males (POR 3.0, 95% CI:1.4–6.7, *p* value = 0.006), those aged ≥50 years (POR 10.8, 95% CI; 4.3–27.3, *p* value < 0.000), and those with family history (POR 3.4, CI; 1.6–6.9, *p* value 0.001) remained associated with diabetes. See Table 5 for the multivariate analysis for factors associated with diabetes prevalence among TB patients, National Tuberculosis Center, Sana’a-Yemen 2021.

## 4. Discussion

Diabetes is an important associated factor for TB complications. TB patients with diabetes mellitus (DM) are almost four times as likely to experience a relapse of their TB after being cured, nearly twice as likely to develop multidrug resistance and twice as likely to die during TB treatment, compared w TB patients without DM [7,11].

In the absence of information on TB–DM comorbidity in Yemen, we conducted this study with the aim of determining the magnitude of diabetes mellitus and its associated factors in TB patients. This study has provided valuable information on the burden and related factors of DM in TB patients, which emphasizes a need for the implementation of interventions.

The results of this study showed a diabetes prevalence rate of 18% among TB patients. This was higher than the results of studies conducted in Ethiopia, 8.3%, and Egypt, 12.4% [18,19]. These differences might be attributable to the differences in study types, which were hospital-based and community-based, respectively.

Compared to other studies, we found that the prevalence of diabetes in our study was lower than the prevalence that has been reported by other studies in Saudi Arabia 27% [20]. One study from India estimated a prevalence of diabetes of up to 29% [12]. The difference in the prevalence in these studies could be due to the differences in the socio-economic and demographic characteristics of the studied population. Likewise, variation in diagnostic methods for DM among the different studies could have affected the reported prevalence.

Our findings showed significantly higher diabetes odds among males compared to females. This finding was similar to a study conducted in Brunei that found a significantly high prevalence among males [21]. Other studies have reported a higher prevalence among females [18,19,22], which might be linked to the influence of estrogen on cytokine production during TB infection leading to an increased susceptibility of women to TB, and consequently to DM [23]. It is worth exploring the causes of this pattern of prevalence variation, taking into consideration the access variables between male and female patients.

Similar to the result of many studies conducted in China [24], Denmark [22], Egypt [19], Nepal and India [12,25], this study showed a higher prevalence among those aged ≥50 years old. This could be explained by the high prevalence of DM in the general population among older people, and may be related to the lower level of immunity of older individuals that makes them more susceptible to developing TB–DM [26].

Diabetes was significantly associated with having a family history of diabetes; this finding was similar to the results of many other studies conducted in other regions [12,18,25], and may be related to familial predisposition due to genetic similarity. There is growing evidence, based on genetic research in some countries, to support this hypothesis. Likewise, a family history of DM was among the factors identified as having an influence on the occurrence of TB–DM comorbidity; having a family history of DM is a known risk factor for DM [27].

Obese patients had significantly higher odds of having DM compared with normal weight patients, whereas both underweight and overweight patients were less likely to have DM. This might be due to the relationship between obesity and DM, and higher proportion of detected DM in our study. It is important to note that excessive weight gain is recognized as a predisposing factor to diabetes and it is an independent risk factor for TB [28]. This result was in contrast with another study in India [29], in which the prevalence was higher among underweight patients due to the common wasting symptoms TB patients, which might be due to different cultural customs and lifestyles.

This study did not find any association between diabetes and type of TB (pulmonary TB), in contrast to the results of other studies that revealed a significant association [12,18,22]. The differences in these studies could be due to the differences in the studied population, as they might have subjects with different age and gender distributions, and treatment durations.

The results of this study did not find any significant differences in the prevalence of diabetes among smokers and khat chewers. This finding is similar to previous studies done in Ethiopia [18], while other studies did find a significant association with behavioral factors such as smoking [12,25]. Differences due to the denial of smoking or khat chewing habits may affect this study’s findings in relation to behavioral factors for DM and TB comorbidity.

The main limitation of this study was that it was conducted in only one TB center; this study’s results could not be generalized since it was a facility-based study conducted only among TB patients who attended the NTC in Sana’a city and did not cover the TB centers in other governorates. Moreover, the inclusion in this study of patients with better survival or less severe disease level could have led to a problematic cause-effect of the associated factors with the disease.

## 5. Conclusions

The prevalence of diabetes and newly identified DM found by screening among diagnosed cases of TB was high. The identified associated factors were gender, males; older age, ≥ 50 years; and a family history of diabetes. To ensure the provision of optimal care for patients with TB–DM, TB patients should be screened for DM immediately after the diagnosis of TB, and should be monitored on a periodic basis during the course of TB treatment. To reduce the dual burden of TB–DM comorbidity, strategic planning for integrated TB–DM services through dual diagnosis should be initiated.

## Figures and Tables

**Table 1:** Sociodemographic and clinical characteristics of TB patients, National Tuberculosis Center, Sana’a-Yemen 2021.

		*n*	%
Sociodemographic characteristics			
Gender	Male	174	53%
	Female	157	47%
Age (year)	<40	192	58%
	40–<60	84	25%
	≥60	55	17%
Place of residence	Rural	173	52%
	Urban	158	48%
Education	Illiterate	147	44%
	Primary	84	25%
	Secondary	69	21%
	University	31	10%
Occupation	Not work	285	86%
	Work	46	14%
Behaviors			
Smoking	No	256	77%
	Yes	75	23%
Chewing khat	No	130	39%
	Yes	201	61%
Clinical characteristics			
Site of TB	Pulmonary tuberculosis	143	43%
	Extra-pulmonary tuberculosis	188	57%
Treatment status	New	247	74%
	Follow up	62	19%
	Relapse	13	4%
	After default	9	3%
Chronic diseases	Heart diseases	47	14%
	Kidney diseases	30	9%
	Liver diseases	4	1%
	Cancer	3	1%
Family history of diabetes	No	233	70%
	Yes	98	30%
BMI	Normal weight (18–22.9 kg/m^2^)	139	42%
	Obesity (>25 kg/m^2^)	43	13%
	Overweight (23–24.9 kg/m^2^)	33	10%
	Underweight (<18 kg/m^2^)	116	35%

**Table 2:** Diabetes status among TB patients at National Tuberculosis Center, Sana’a-Yemen 2021.

Characteristics	*n*	%
Diabetes statues (*n* = 331)		
Diabetic	58	18%
Non-Diabetic	273	82%
Known diabetes (*n* = 58)		
Yes	34	11%
No	22	6%
Do not know	2	1%

**Table 3:** Characteristics of TB–DM with TB patients, National Tuberculosis Center, Sana’a-Yemen 2021.

Category	TB–DM (*n* = 58)	TB only (*n* = 273)
Sociodemographic characteristics:	*n* (%)	*n* (%)
Gender		
Female	19 (33)	138 (51)
Male	39 (67)	135 (49)
Age group by year		
<50 years	20 (34)	218 (80)
≥50 years	38 (66)	55 (20)
Place of residence		
Rural	30 (52)	143 (52)
Urban	28 (48)	130 (48)
Education		
Yes	23 (40)	161 (59)
No	35 (60)	112 (41)
Occupation		
Work	8 (14)	38 (14)
Not work	50 (86)	235 (86)
Behavioral characteristics:		
Smoking		
No	42 (72)	214 (78)
Yes	16 (28)	59 (22)
Chewing khat		
No	23 (40)	107 (39)
Yes	35 (60)	166 (61)
Clinical characteristics:		
Site of TB		
Pulmonary tuberculosis	26 (45)	117 (43)
Extra-pulmonary	32 (55)	156 (57)
Chronic diseases		
No	38 (66)	213 (78)
Yes	20 (34)	60 (22)
Family history of diabetes		
No	32 (55)	201 (74)
Yes	26 (45)	72 (26)
Nutritional status:		
BMI		
Normal	24 (41)	115 (42)
Obesity	14 (24)	29 (11)
Overweight	9 (16)	24 (9)
Underweight	11 (19)	105 (38)

**Table 4:** Univariate analysis for factors associated with diabetes prevalence among TB patients, National Tuberculosis Center, Sana’a-Yemen 2021.

Category	Crude POR * (95% CI **)	*p* Value
Sociodemographic characteristics:		
Gender		
Male vs. Female	2.1 (1.2–3.8)	0.014
Age group by year		
≥50 years vs. <50 years	7.5 (4.1–14.1)	0.000
Place of residence		
Urban vs. Rural	1.0 (0.6–1.7)	0.927
Education		
No vs. Yes	2.2 (1.2–3.9)	0.007
Occupation		
Not work vs. Work	1.0 (0.5–2.3)	0.979
Behavioral characteristics:		
Smoking		
Yes vs. No	1.4 (0.7–2.6)	0.323
Chewing khat		
Yes vs. No	1.0 (0.5–1.8)	0.947
Clinical characteristics:		
Site of TB		
EPTB vs. PTB	1.1 (0.6–1.9)	0.783
Chronic diseases		
Yes vs. No	1.9 (1.0–3.5)	0.043
Family history of diabetes		
Yes vs. No	2.3 (1.3–4.1)	0.005
Nutritional status:		
BMI		
Obesity vs. Normal	2.3 (1.1–5.0)	0.031
Overweight vs. Normal	1.8 (0.7–4.3)	0.189
Underweight vs. Normal	0.5 (0.2–1.1)	0.072

* POR = Prevalence odds ratio; ** CI = 95% confidence interval.

**Table 5:** Multivariate analysis for factors associated with diabetes prevalence among TB patients, National Tuberculosis Center, Sana’a-Yemen 2021.

Category	Crude POR (95% CI)	*p* Value	Adjusted POR (95% CI)	*p* Value
Sociodemographic characteristics:				
Gender				
Male vs. Female	2.1 (1.2-3.8)	0.014	3.0 (1.4-6.7)	0.006
Age group by year				
≥50 years vs. <50 years	7.5 (4.1-14.1)	0.000	10.8 (4.3-27.3)	0.000
Family history of diabetes				
Yes vs. No	2.3 (1.3-4.1)	0.005	3.4 (1.6-6.9)	0.001

## Data Availability

All relevant data presented in this paper, and more information can be provided upon reasonable request from the corresponding author.
